# Automated Medical Care: Bradycardia Detection and Cardiac Monitoring of Preterm Infants

**DOI:** 10.3390/medicina57111199

**Published:** 2021-11-03

**Authors:** Beatrice Arvinti, Emil Radu Iacob, Alexandru Isar, Daniela Iacob, Marius Costache

**Affiliations:** 1 Fundamentals of Physics for Engineers Department, “Politehnica” University Timisoara, Bd. Vasile Pârvan 2, 300223 Timisoara, Romania; beatrice.arvinti@upt.ro; 2 Department of Pediatric Surgery, “Victor Babes” University of Medicine and Pharmacy, Eftimie Murgu Square 2, 300041 Timisoara, Romania; radueiacob@umft.ro; 3 Faculty of Electronics, Telecommunications and Information Technologies, “Politehnica” University Timisoara, Bd. Vasile Pârvan 2, 300223 Timisoara, Romania; alexandru.isar@upt.ro; 4 Department of Neonatology, “Victor Babes” University of Medicine and Pharmacy, Eftimie Murgu Square 2, 300041 Timisoara, Romania; danielariacob@yahoo.com

**Keywords:** remote monitoring, preterm birth, electrocardiogram, arrhythmia

## Abstract

*Background and Objectives*: Prematurity of birth occurs before the 37th week of gestation and affects up to 10% of births worldwide. It is correlated with critical outcomes; therefore, constant monitoring in neonatal intensive care units or home environments is required. The aim of this work was to develop solutions for remote neonatal intensive supervision systems, which should assist medical diagnosis of premature infants and raise alarm at cardiac abnormalities, such as bradycardia. Additionally, the COVID-19 pandemic has put a worldwide stress upon the medical staff and the management of healthcare units. *Materials and Methods*: A traditional medical diagnosing scheme was set up, implemented with the aid of powerful mathematical operators. The algorithm was tailored to the infants’ personal ECG characteristics and was tested on real ECG data from the publicly available PhysioNet database “Preterm Infant Cardio-Respiratory Signals Database”. Different processing problems were solved: noise filtering, baseline drift removal, event detection and compression of medical data using the à trous wavelet transform. *Results*: In all 10 available clinical cases, the bradycardia events annotated by the physicians were correctly detected using the RR intervals. Compressing the ECG signals for remote transmission, we obtained compression ratios (CR) varying from 1.72 to 7.42, with the median CR value around 3. *Conclusions*: We noticed that a significant amount of noise can be added to a signal while monitoring using standard clinical sensors. We tried to offer solutions for these technical problems. Recent studies have shown that persons infected with the COVID-19 disease are frequently reported to develop cardiovascular symptoms and cardiac arrhythmias. An automatic surveillance system (both for neonates and adults) has a practical medical application. The proposed algorithm is personalized, no fixed reference value being applied, and the algorithm follows the neonate’s cardiac rhythm changes. The performance depends on the characteristics of the input ECG. The signal-to-noise ratio of the processed ECG was improved, with a value of up to 10 dB.

## 1. Introduction

Prematurity of birth occurs before the 37th week of gestation, affecting up to 10% of births worldwide [1,2]. The European average of preterm birth rate is 7.3%, and the percentage of babies with low birth weight (lower than 2500 g) varies between 4.5% and 8% of all births [3]. Prematurely born infants and neonates with low birth weight are associated with an immature cardio-respiratory system and an immature immune system, being at greater risk of developing severe infections [4,5,6]. Additionally, the COVID-19 pandemic with the associated severe acute respiratory syndrome coronavirus 2 (SARS-CoV-2) puts some stress on both mother and child, with many questions still being left about the future development of the newborn. The psychological stress upon the mother should not be neglected [7,8], as several studies have associated SARS infections during pregnancy with preterm delivery and intrauterine growth restrictions [9,10,11]. The pandemic is also reported to affect the work of neonatal intensive care unit (NICUs) [12]. The latest developments in the SarsCoV-2 virus affects many children (according to CNN, “Hospitalizations were highest among kids aged up to 4, and teens aged 12–17. One in four of the children who were hospitalized needed intensive care.” [13]): lately (19 October 2021), Romania has also reported a record number of COVID infections of 18,863 cases [14], with 486 infected minors. Long-term monitoring of newborns is recommended to establish better the implications of the novel coronavirus [7,10]: remote supervision and automatic diagnosis algorithms aim to provide access to medical assistance in times when hospitals worldwide are under stress and lack sufficient medical personnel. Remote monitoring of neonates is not an easy task, as the newborns are usually moving and various interferences add noise to the useful ECG signal. Technical solutions have been developed to aid medical diagnosis [15,16,17] and enable a fast clinical reaction. Another management solution consists in the transfer of some of responsibility for medical care to children’s parents through remote home monitoring [18,19,20]. Additionally, infants are able to be infected with the coronavirus at a later date: their immune system is less developed, and therefore neonates need careful supervision [21]. 

The preterm state is a vulnerable state, highly related to sudden infant death syndrome (SIDS), due in part to apnea and sleep disorders, calling for special sleep practice to ensure an infant’s safety [22,23,24,25]. Sleep-related cardio-respiratory instabilities are particularly important to infants, as preterm newborns spend up to 70–90% of the day sleeping. The risk of cardiovascular instabilities is marked during sleeping phases [22]. Respiratory pauses (called apnea-bradycardia episodes), associated with heart rhythm disorders, are stated as being common to preterm infants [26,27,28]. Bradycardia is defined as a cardiac event, where the heart rate slows to less than 100 bpm for at least two beats in duration [29] (in terms of RR-peaks, the time interval between the consecutive R-peaks must be >0.6 s). Fetal distress is reflected in physiological changes that can be outlined by changes in heart rate variability (HRV) [30]. Real-time monitoring of HRV metrics are linked [31,32,33] to information about the neurological development of the infant: newborns with higher root mean square of successive RR-interval differences (RMSSD), standard deviation of normal RR-intervals (SDNN) and standard deviation of the average normal RR-intervals (SDANN) showed a better neurological development [31,33]. The relation of RMSSD to the infant’s vagal activity could help clinicians in interpreting RMSSD changes: the lower the RMSSD, the lower the modulation by the vagal activity, worsening the infant’s condition [31]. Still, a sudden rise of RMSSD value can be an indicator of other pathological conditions, such as an intrapartum hypoxia-ischemia (reduced oxygen supply) leading to brain injury [31,34,35] (in adults, low HRV is associated with an increased risk of coronary heart disease and a predisposition to premature heart attacks [36,37]). As a study conclusion, the more altered the HRV metrics are, the worse the clinical outcome is (leading to brain injury or even SIDS) [38]. Personalized and automated diagnostic algorithms should relieve medical stress and assist clinical diagnosis. Some studies associate cardiac arrhythmias (such as bradycardia) with clinical post-symptoms of COVID: bradycardia and COVID-19 have been brought together in recent studies [39,40,41,42,43,44,45,46,47,48,49,50,51], with an increased incidence of cardiovascular symptoms in patients (both adults and children) being reported. Thus, an automatic surveillance system (for NICU or home monitoring) has a practical medical application. There are also future plans to implement the software algorithms on a hardware device and to build a prototype for a portable monitoring system for neonates, based on Arduino, enabling additional clinical studies on the occurrence and effects of cardiac arrhythmias in infants.

## 2. Materials and Methods

The authors tested the signal processing algorithms on the recordings provided by PhysioNet [28,52,53], managed by members of the MIT Laboratory for Computational Physiology. The Research Resource for Complex Physiologic Signals was established in 1999 under the auspices of the National Institutes of Health (NIH). The ECG database for preterm infants (added in 2017) can be downloaded as “Preterm Infant Cardio-Respiratory Signals Database” from the physionet.org website [52,53]. The ECGs were recorded at 250 Hz (*infant1_ecgm* and *infant 5_ecgm*) and at 500 Hz, respectively, and were recorded for 10 subjects in the NICU at University of Massachusetts Memorial Healthcare [28]: infant1 presented a post-conceptional age (PCA) of 29 weeks, with a birth weight of 1200 g. During 45.6 h of recording, 77 bradycardia episodes were reported. Infant2 and infant3 presented a PCA of 30 weeks and 5 days, birth weights of 1760 g and 1710 g, respectively, and during the 43 h of recording, a number of 72 and 80 bradycardias were reported. Infant4 presented a PCA of 30 weeks and a low birth weight (840 g). During 46.8 h of recordings 66 bradycardias were reported. Infant5 presented a PCA of 32 weeks, a birth weight of 1670 g, and 72 bradycardia episodes during 48.8 h of recording. Infant6, infant7 and infant9 reported a PCA of 30 weeks and similar birth weights: 1140 g, 1110 g and 1230 g, with 56, 34 and 97 respective bradycardia episodes being detected during recording. The high number of bradycardias detected in infant9 can also be correlated with the increased recording time (70.3 h of recording). Infant8 and infant10 presented the highest PCA (32 weeks and 34 weeks, respectively) and the highest birth weights (2100 g and 1900 g, respectively). A number of 28 and 40 bradycardias, respectively, were reported during recording. The infants were breathing room air and did not show perinatal or congenital infection of the CNS or hypoxic-ischemic encephalopathy [28,53].

**Figure 1:**
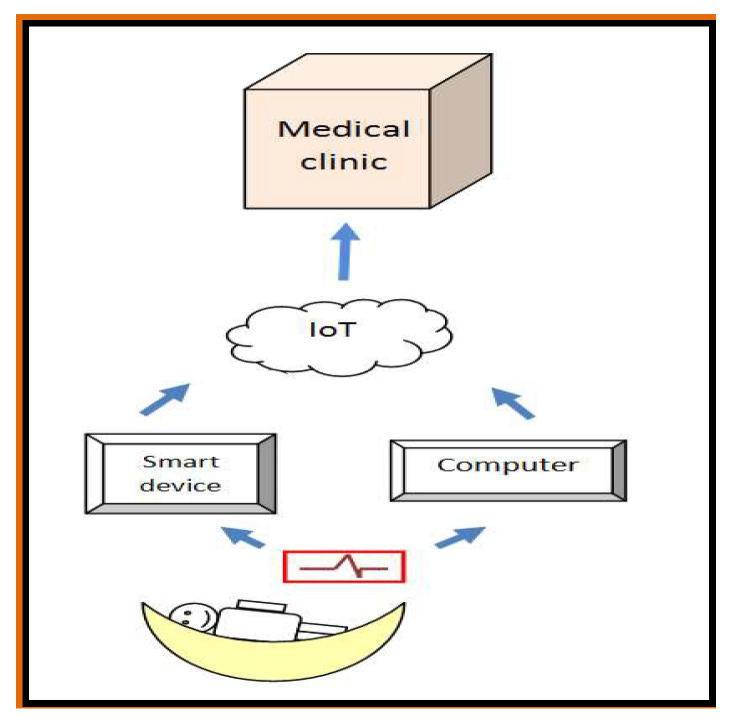
Monitoring architecture.

We aimed to develop a portable device for remote monitoring of newborn infants that would ease the work of overcrowded hospitals in times of the pandemic. For non-invasive monitoring, we proposed a scenario (Figure 1) intended to alert both NICU clinicians and parents of the infant to an intervention necessity. The cardiac activity of the infant could be monitored through a wearable ECG device [54,55] and the signals would be transmitted via Bluetooth to either a NICU computer or a mobile device of the parent. Mobile devices and computers would perform elementary tasks such as data collection, data management and filtering, compression and primary interpretation of ECG rhythm (normal sinus rhythm or arrhythmia). The data would be transmitted via Internet of Things (IoT, used to create a home network to connect and exchange data with a remote monitoring device, for example) or Cloud to a central medical data record unit for final interpretation.

**Figure 2:**
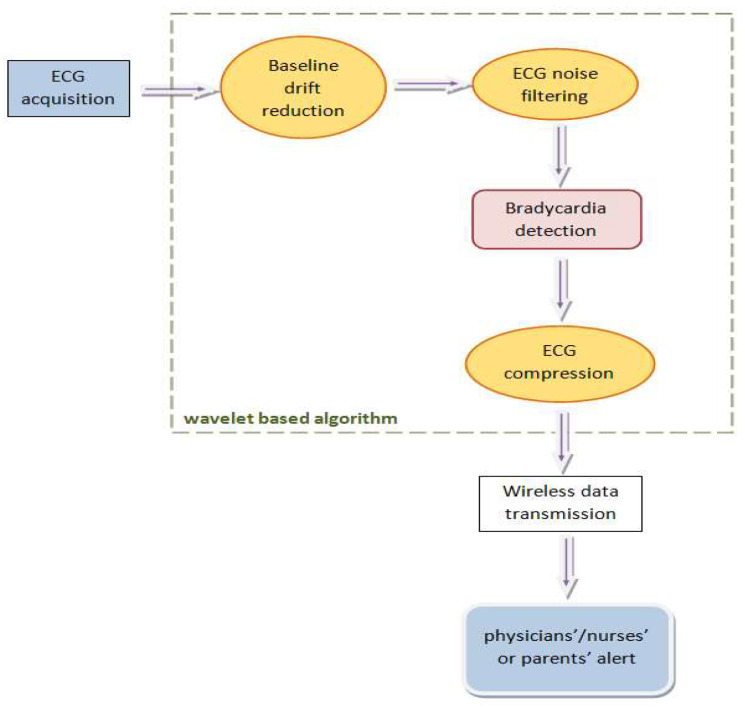
Structure of the automated monitoring system.

The working scheme of the proposed algorithm was constructed using the same wavelet transform for all processing steps (Figure 2), to exploit the properties of time-frequency analysis with minimal resources. No fixed threshold was defined; thus, the algorithm was personalized for each newborn. A geometric measure was defined—the relative RR intervals (RR_k_) [56]. This interval defines the relative variation of two neighboring R-peaks in time, where k is the number of RR-interval terms. Medical engineering applications need a quantification of physical quantities, which would allow us to model and predict a clinical development. For example, the RR_k_ value should differ only to a small amount (approximately between −20% and +20% [56]), as a higher value would indicate a high irregularity of the RR-intervals (a first symptom of a cardiac abnormality). We also tried to avoid standardized values, as gestational age of preterm infants is usually different, and we consider that each medical interpretation should be related to the infants’ characteristics. The study focused on designing signal processing algorithms for a preliminary diagnosis and arrhythmia alarm—especially if remote monitoring of the newborn would be realized at home due to a lack of hospital space (because of the outbreak of the COVID-19 pandemic). RR_K_ should also enable us to detect a preterm infant’s cardiac status, such as apnea-induced bradycardia (1):

RR_k_ = 2(RR_k_ − RR_k−1_)/(RR_k_ + RR_k−1_) (1)

$$\{\begin{array}{c} {\mathrm{RR}}_{k}>0.6\;\;s\;\;\;\;\;\;\;\mathrm{BRADYCARDIA}\;\;\;\;\mathrm{ALERT}\\ {\mathrm{RR}}_{k}<0.6\;\;s\;\;\;\;\;\;\;\mathrm{Normal}\;\;\mathrm{Sin}\mathrm{us}\;\;\mathrm{Rhythm} \end{array}$$

Daily life signals reside in the time domain, still sometimes full information about the signal is not always visible. Thus there is a necessity to transform the signal from the time domain to other domains (such as the frequency domain [30]) to enable computer-assisted solutions. ECGs trace the variation of a voltage over time, with no information about the frequencies that might appear during the signal acquisition (a non-visible information). Wavelets check the frequency content of the signal at multiple resolutions of the analyzed signal.

Wavelets are mathematical tools that allow us to assess the intervention on several frequencies: we can transform a signal in the frequency domain and check if there are frequencies that should not appear in cardiac signals (ECG frequencies are usually between 0.6 and 40 Hz). Other frequencies indicate external interference/noise (for example, 50–60 Hz interference is due to a nearby power line) and should be rejected to obtain a neat signal. The useful information can thus be extracted (Equation (2)) and the signals can be returned to the time domain after processing. Wavelets analyze the desired content of the ECG using multiple resolutions of the signal so as to adjust the level of detail needed [57,58,59]. Wavelets go from a larger scale to a smaller one (but with more details) through the shifting and dilating of a basic function called the mother wavelet *ψ_a_*_,*b*_ (MW). MW is selected in accordance with the analyzed signal (we are actually zooming on the studied signal), Equation (2):

(2) $$W_{\psi }s(a,b)=\langle s(t),\psi_{a,b}(t)\rangle =\;\int_{-\infty }^{\infty }s(t)\cdot \frac{1}{\sqrt{a}}\psi^{*}(\frac{t-b}{a})dt,\;\;\;\;\;\;\;a\in R,\;\;b>0$$

where the wavelet transform (WT) of the analyzed signal *s*(*t*) is obtained through scaling and translating the function called the mother wavelet *ψ_a_*_,*b*(*t*)_, using dilation (*a*) and translation (*b*) parameters. The asterisk * denotes the complex conjugate of the function.

**Figure 3:**
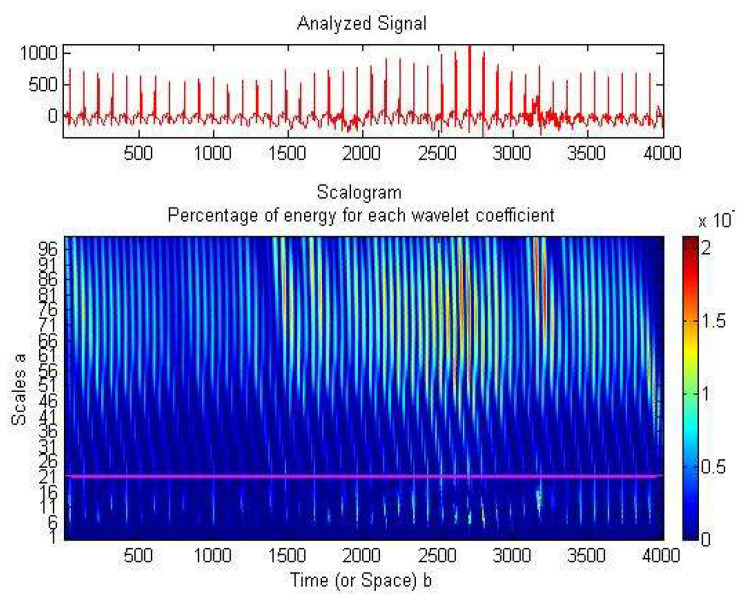
Energy percentage for the wavelet coefficients, using db8 mother wavelet. Analyzed signal waveform (first panel) and its scalogram (second panel).

Baseline drift reduction (due to infant movements), noise filtering and ECG compression might use wavelets’ property of sparsity [60,61]: the signal’s energy will be focused into a small amount of non-zero wavelet coefficients. The frequency domain augments the knowledge about the analyzed signal (Figure 3). We noticed an even distribution of all signal samples and a concentration of the wavelet coefficients’ energy on the higher scales, corresponding to low frequencies (up to 20 Hz, with a peak at 15.62 Hz). Thus, we adapted the WT on the lower frequencies so as to concentrate the signal and to reduce as many zero wavelet coefficients as possible. This resulted in a neater and more compressed signal. Suited to our necessities was a translation invariant transform, such as the stationary wavelet transform (SWT), implemented with an algorithm known as “algorithme à trous” [62]. SWT passes the signal successively through a series of low-pass filters (computing the approximation wavelet coefficients cA) and high-pass filters (computing the detail wavelet coefficients cD). Using these coefficients, several signal processing tasks were performed:

### 2.1. Baseline Drift Removal

To reduce the baseline drift, we approximated the general tendency of the ECG signal using only the wavelet approximation coefficients, on 5 iteration levels.

### 2.2. Denoising

The signal was filtered using an adaptive algorithm in the wavelet domain and a simple soft-thresholding filter. The filter was applied on the detail wavelet coefficients (they corresponded to the high frequency components of the ECG signal). The threshold was related to the small values of the detail wavelet coefficients, which contained the artifacts. Detail coefficients were different for each patient, but were always low in value. We can not reconstruct the ECG eliminating all detail wavelet coefficients, but we eliminated the lower values between them to obtain a neater signal. Therefore, any wavelet coefficient lower than the established threshold was rejected as noise. The method was personalized on the ECG characteristics of each supervised infant (3) and could thus diagnose HRV changes of each infant.

(3) $$\begin{array}{l} \mathrm{for}\;\;i=1\;\mathrm{to}\;k\\ T_{\mathrm{ECG}\mathrm{infant}}=\sqrt{\frac{1}{n-1}\cdot \sum_{k}{{(\mathrm{cD}}_{i}{-\mathrm{mean}(\mathrm{cD}}_{i}))}^{2}\;} \end{array}$$

where *i* is the number of the WT decomposition level, *k* the total number of WT iterations, cD the wavelet detail coefficient and *n* is the ECG sample length.

### 2.3. Preterm Infant ECG Bradycardia Detection

To personalize the algorithm so as to suit the need of each monitored infant, the R-peak value was also adaptively set: the maximal value of the ECG recording (the R-peak), was computed after a delay of 2 s, to cover at least one heartbeat. Values lower than 70% of the infants’ reference peak were rejected and only the peak values were retained. We needed to define a tolerance, as no medical parameter has the same value at all measurement times, still there were no major clinical drawbacks. Thus, we established a threshold of 70% of the reference value to account for a slight baseline drift of the newborn, as newborns are moving and electrodes might slip slightly. After the detection of two heartbeats, the RR-interval was computed and stored. An alarm was set if the time delay between two neighboring RR-peaks > 0.6 s, and the time interval was stored to enable further investigations.

### 2.4. Data Compression

Compression ratio (CR) is a performance estimator for the compression of a signal (we want the ECG signal to occupy less computer memory, to have a longer cardiac monitoring over time). Continuous monitoring resulted in a large quantity of data; therefore, engineering solutions that achieved a minimal loss of clinical information were searched for.CR refers to the ratio between the number of input bits related to the number of output bits. Signal compression can be realized using a number of fewer bits for each iteration level, rejecting the zero-valued wavelet coefficients. The final signal was reconstructed on a minimal number of bits, achieving a compression ratio that depended on the number of iterations used [61]. 

## 3. Results

**Figure 4:**
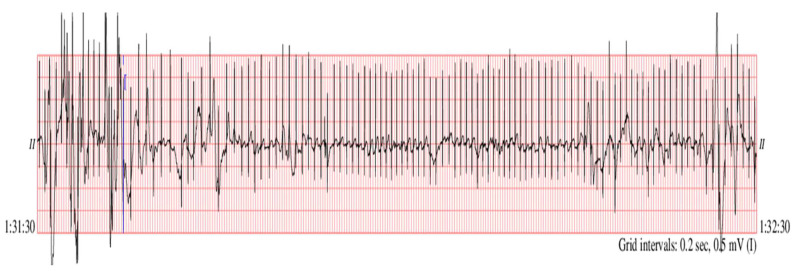
Original ECG named *infant6_ecgm*, after 1 h, 31 min and 30 s of standard recording (https://archive.physionet.org/cgi-bin/atm/ATM (accessed on 29 August 2021).

**Figure 5:**
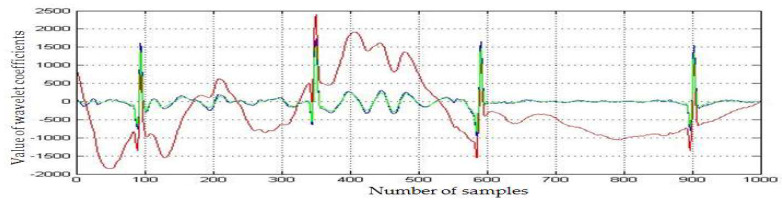
Superposition of original ECG (red color), ECG with reduced baseline drift (blue color) and denoised ECG (green color), for *infant6_ecgm*.

A noisy segment of the recording named *infant6_ecgm* (time of recording 1 h 31 min 30 s:1 h 32 min 30 s) was taken to check two clinical aspects (Figure 4): whether the baseline drift removal algorithm is efficient on segments with pronounced drift and whether the mentioned algorithm was influencing the detection of bradycardia segments which occurred on noisy ECG segments. The baseline drift removal (Figure 5, blue color) and the denoising procedure (Figure 5, green color) improved the SNR, and we also noticed the correction of the baseline drift. We obtained a high SNR improvement of 10.18 dB.

For the analyzed preterm infants’ database, the SNR improvement varied between 1.34 and 10.18 dB.

**Figure 6:**
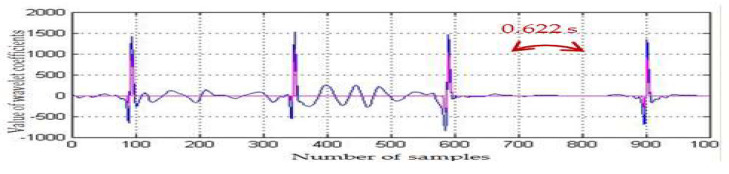
*infant6_ecgm* (hour 1.31.36–1.31.38 of recording).

**Figure 7:**
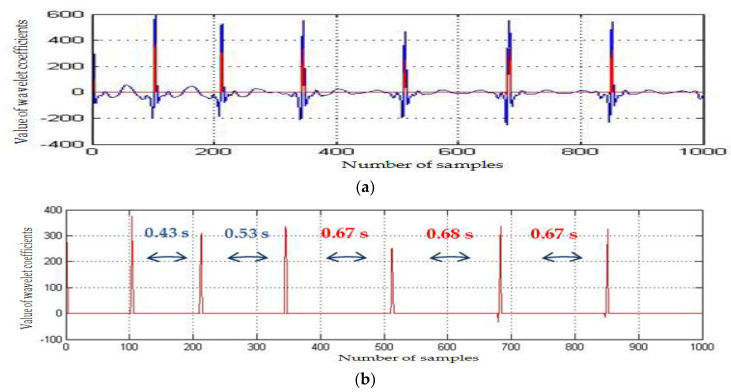
(**a**) Zoom on bradycardia event for *infant1_ecgm*, db8 MW, 1000 samples (minute 32–33 of recording). (**b**) Zoom on the detected RR-intervals for *infant1_ecgm*, db8 MW, 1000 samples (minute 32–33 of recording).

Accurate alarm on bradycardia episodes was realized by detecting the R-peaks of the ECG segments and computing the RR-intervals (Figure 6 and Figure 7). To keep the processing time delay as minimal as possible and to allow a continuous monitoring and a timely detection of cardiac anomalies, we operated on blocks of 1000 samples: 1000 samples were processed, while the next 1000 samples were stored. The authors applied the algorithm to the different ECGs of infant recordings available on PhysioNet and the results are synthesized in Table 1. Thus, for the analyzed ECGs, we achieved varied compression ratios (CR): between 1.72 (*infant5_ecgm*) and 7.42 (*infant6_ecgm*), using an 8-bit resolution. The performance (CR, SNR improvement) depended on the characteristics of the input ECG.

The SNR of the processed ECG was improved with a median value of 4 dB: the higher the value of the dB, the stronger the useful signal and the weaker the noise.

**Figure 8:**
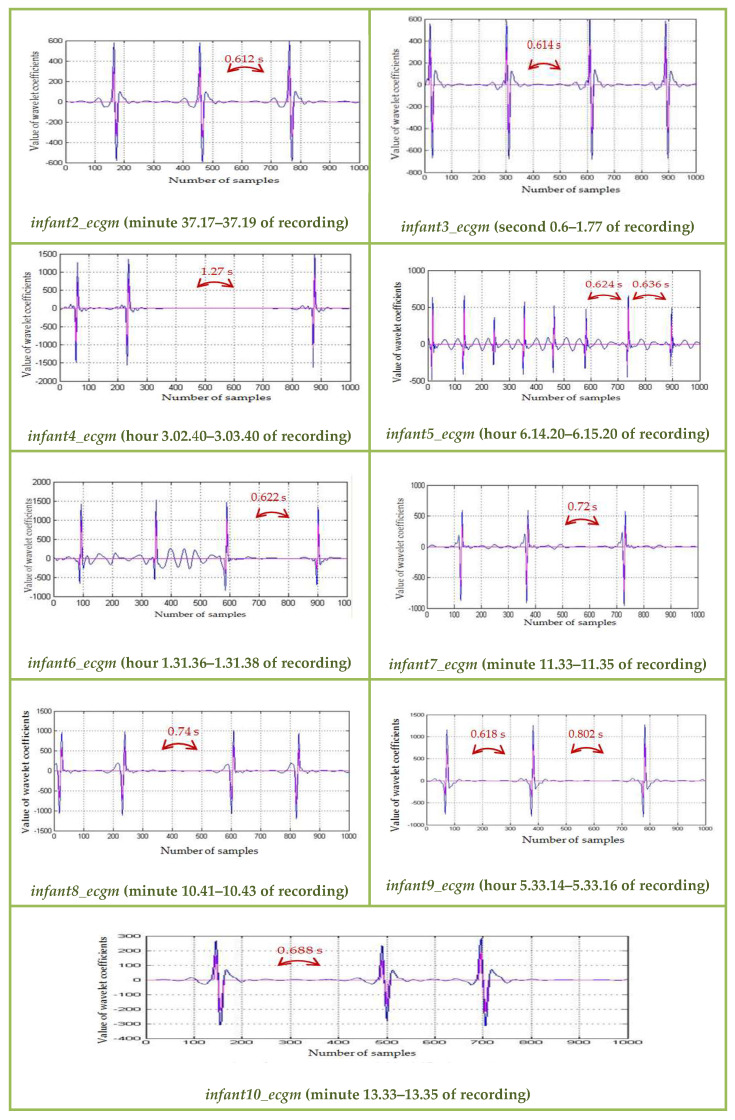
Zoom on the detected R-peak values on bradycardia events for preterm infants of the PhysioNet database, db8 MW, 1000 samples.

The CR reflected the characteristics of the ECG recording and can be further enhanced through lossy compression procedures. The proposed compression procedure was personalized, with the CR depending on the regularity of the preterm ECG signal: ECGs with higher baseline drift achieved higher CRs (as an example, for ECG named *infant6_ecgm*, we reached a high CR of 7.42). A total of 1000 samples segments for all studied cases are displayed in Figure 8. The bradycardia alarm given through the proposed method coincided with the annotated bradycardia episodes on the PhysioNet database [52].

The processing time for a sample length of 2 s (1000 samples) was less than 1 s for a standard Intel Core2 computer, on 1.86 GHz and 4.00 GB RAM memory. The samples were digitized on a reduced number of bits and transmitted via Bluetooth connection or IoT to a central NICU or smart device for storage and further processing.

## 4. Discussion

The SarsCoV-2 virus affects both adults and children. The medical crisis due to the COVID-19 pandemic determined us to find solutions for remote monitoring of vital signs. Remote monitoring of neonates is not an easy task, as newborns are usually moving, and various interferences add noise to the useful ECG signal. The algorithm aimed to relieve the stress put on a NICU, where prematurely born infants need constant supervision, developing a method to automatically supervise the cardiac monitoring of newborns. The algorithm relies on the traditional diagnostic scheme, but it is implemented with the aid of powerful mathematical operators. The solutions should be implemented on portable devices and should also enable data acquisition and intervention in home environments. The algorithm is tailored to an infant’s personal ECG characteristics. To avoid errors due to infant movements, which are reflected in drifts of the ECG baseline and higher R-peak values, we applied a baseline drift correction method after a minimal delay of 2 s from acquisition start. There is a necessity to correct the baseline drift for accurate automated peak identification. As can be noticed analyzing Figure 5, Figure 6 and Figure 7, the baseline drift was completely suppressed. The sample was filtered and a thresholding algorithm was applied in the time domain, detecting the R-peaks. Considering the filtering procedure, in [63] and [64] are proposed denoising methods based on the discrete wavelet transform (DWT). The DWT is a non-redundant, shift variant transformation, which has poor denoising performance, although a smaller redundancy than the SWT. DWT is a translation variant transform (contrary to SWT): the signal is passed through a series of low-pass (allowing low frequency components) and high-pass filters (allowing high frequency components). At the output of each filter, DWT eliminates every second coefficient, resulting in fewer and fewer coefficients if the signal analysis is done in more steps. The results will be then constructed through interpolation (approximations of the signal), and some information might be thus lost. This procedure was not applied in case of the SWT (there was no downsampling by a factor of 2 realized), and thus SWT allowed a much more accurate signal reconstruction. Using a denoising procedure in three steps, with the DWT and a fixed threshold value searching method, the authors of [63] also conducted experiments using only a set of simulated noisy ECG signals. The MW used for DWT computation was not specified. The authors of [63] did not take into account important aspects of real ECGs, as, for example, the baseline drift, making difficult an objective comparison with our results shown on the fourth column of Table 1. In [64], the same denoising method as in [63] was studied, but different MWs and different filtering procedures in the wavelet domain were considered. The authors of [64] compared the soft-thresholding filter (selected by us as well) with the hard-thresholding filter and took into account four strategies for the selection of a fixed threshold value: universal, rigorous SURE, heuristic SURE and the minimax criterion. They prove the superiority of soft-thresholding filter versus the hard-thresholding filter from the SNR improvement point of view. The authors of [64] found that the best MWs for the filtering of the DWT wavelet coefficients with the soft-thresholding filter were sym4 and coif8. The fact that for baseline correction and for noise filtering there are different MWs is a drawback of the DWT. We estimated, based on Figure 8 in [64], the SNR enhancement of the DWT-based denoising method. The best value of 5 dB was obtained for an input SNR of 0 dB. The worst value of the SNR enhancement obtained, using DWT of 1.4 dB, was obtained for an input SNR of 8 dB. The mean value of the SNR enhancement, obtained for input SNRs between 0 dB and 8 dB, equaled 3.6 dB. Our mean value of the SNR improvement (displayed in Table 1) was of 4.2 dB. Therefore, the use of SWT computed with the MW db8 and the adaptive selection of the threshold following equation (3) made our denoising method better. For R-peaks detection, [65] proposed a different method. First the SWT using the quadratic spline MW is computed, and next the wavelet modulus maximum algorithm is applied. To detect R-peaks, this method requires the intervention of experts. Hence, contrary to the proposed R-peaks detection, the method in [65] is not automated.

In our study, the-RR interval between two neighboring samples was computed: if the interval was >0.6 s, an alarm was raised, calling for the necessity of further investigations. Additionally, a sudden rise of RMSSD could be an indicator of pathological conditions, such as a reduced oxygen supply (which might lead to brain injury). We propose the computing of HRV metrics (for example, the RMSSD) for every 2 s or 5 s to check the personal cardiac variation of neonates.

## 5. Conclusions

The COVID-19 pandemic has placed an additional stress on intensive care units, as preterm children are prone to infections due to their immature immune system. Remote monitoring enables a timely intervention, when there is lack of medical personnel and restricted or no healthcare access of the infants’ relatives because of the pandemic. We tried to offer a solution for long-term unsupervised monitoring, which should be able to alert medical personnel in the event of cardiac rhythm changes, such as bradycardia. Analyzing the preterm ECG database available on PhysioNet, we noticed that a significant amount of noise could be added to a signal while monitoring using standard clinical sensors. Additionally, recent studies have shown that persons infected with the COVID-19 disease are reported to develop cardiovascular symptoms and cardiac arrhythmias. An automatic surveillance system (both for neonates and adults) has thus a practical medical application. The displayed figures (Figure 5, Figure 6, Figure 7 and Figure 8) state that the algorithm detected time durations >0.6 s, which were correlated with bradycardia episodes. The authors tested whether the same wavelet transform could be used and adapted to perform several processing steps (baseline drift removal, denoising, compression, data transmission). We arrived at the conclusion: (1) that we need a translation invariant transform and (2) compression performance is related to the noise contained in the original signal (the greater the removed noise, the higher the compression ratio). A novel mother wavelet tailored to ECG characteristics can be a future interesting research direction. Several signal processing steps were performed with the same mathematical operator (SWT) to keep the operations to a reduced level of complexity. We aimed to allow further development and implementation of the algorithm on a standard smartphone device. The algorithm can be personalized, following the neonate’s cardiac rhythm changes.

The bradycardia events annotated by physicians were correctly detected, as can be noticed in Figure 6, Figure 7 and Figure 8. Additionally, as we take into consideration the distance between RR-peaks, alarms can also be easily triggered for tachycardia: we must set the system to alert medical personnel if the RR-interval is too great for children (>140 beats per minute, or two detected beats >0.43 s). We tested the ECG algorithms to ensure that we have a working basis for the future development of a remote monitoring system for neonates. Future aims are to test the algorithm on neonates from Timisoara, to provide more clinical characteristics of the studied population. There are still open possibilities of exploring the information contained by a signal: an investigation to be undertaken is to verify whether respiration samples of the preterm infant can be influenced by viruses such as SARS-CoV-2, and whether these changes can be highlighted while analyzing the signals with wavelets.

## Figures and Tables

**Table 1:** Performance of RR peak detection wavelet algorithm for the preterm infants ECGs.

ECG	Detected RR-Interval > 0.6 (s) (1000 Samples)	CR Values (1000 Samples)	Statistical Parameters	
			SNR Improvement (dB)	RMSSD (ms) (1000 Samples)
*infant1_ecgm*	0.67, 0.68, 0.67	2.57	4.07	76.48
*infant2_ecgm*	0.612	3.07	1.34	18.38
*infant3_ecgm*	0.614	6.59	4.95	42.58
*infant4_ecgm*	1.27	3.79	2.84	654.38
*infant5_ecgm*	0.624, 0.636	1.72	6.42	60.56
*infant6_ecgm*	0.622	7.42	10.18	82.67
*infant7_ecgm*	0.72	2.92	1.69	165.46
*infant8_ecgm*	0.74	2.99	1.86	249.89
*infant9_ecgm*	0.618, 0.802	4.44	3.71	130.107
*infant10_ecgm*	0.688	5.30	4.91	195.161

## Data Availability

The anonymized preterm infants’ ECG datasets have been takenfrom PhysioNet (https://archive.physionet.org/ accessed on 31 October 2021), which offers free web access to collections of recorded physiological signals. The “Preterm Infant Cardio-Respiratory Signals Database (picsdb)” can be accessed at the link: https://archive.physionet.org/cgi-bin/atm/ATM (accessed on 31 October 2021). Additional information and DOI for the “Preterm Infant Cardio-Respiratory Signals Database”: doi:10.13026/C2QQ2M (https://archive.physionet.org/physiobank/database/picsdb/ accessed on 31 October 2021).
